# Knowledge of the Glasgow Coma Scale among Nurses in a Tertiary Hospital in Ghana

**DOI:** 10.1155/2019/5829028

**Published:** 2019-06-24

**Authors:** Afizu Alhassan, Abdul-Ganiyu Fuseini, Ajara Musah

**Affiliations:** ^1^Nursing and Midwifery Training College, Kpembe, P.O. Box SL98, Salaga, Ghana; ^2^Department of Nursing, School of Allied Health Sciences, University for Development Studies, P.O. Box 1883, Tamale, Ghana; ^3^Neurosurgical Ward, Tamale Teaching Hospital, P.O. Box 16, Tamale, Ghana

## Abstract

**Background:**

Knowledge of the Glasgow Coma Scale (GCS) is recognized as an asset to all clinical nurses. However, many studies in different countries have reported low levels of knowledge of the GCS among nurses. Little is known about this subject in Ghana.

**Objectives:**

The aim of this study was to assess the knowledge of Ghanaian nurses about the Glasgow Coma Scale and identify factors associated with their knowledge.

**Method:**

This was a descriptive cross-sectional study involving a convenience sample of 115 nurses from a large teaching hospital in Ghana. We collected data using a structured questionnaire and analysed the data using descriptive statistics, Pearson's correlation, independent samples t-test, and one-way ANOVA.

**Results:**

A little more than half of the participants (50.4%) had low knowledge of the GCS as a whole. However, with respect to basic theoretical concepts of the GCS, 62.6% of the participants had good knowledge about it, while only 5.2% demonstrated good knowledge on application of the basic knowledge in clinical scenarios. Working in Neurosurgical ward, female gender, and weekly performance of the GCS were associated with higher levels of knowledge. Academic qualification, years of experience as a nurse, and refresher training on GCS were not associated with knowledge.

**Conclusion:**

The findings from this study showed that nurses in Ghana have low levels of knowledge about the GCS. A more structured approach to teaching the GCS that is very thorough and done with demonstrations should be implemented to improve nurses' knowledge on the GCS.

## 1. Background

In 1974, two professors of neurosurgery at the Institute of Neurological Sciences, University of Glasgow, Graham Teasdale and Bryan J. Jennett, developed a tool known as the Glasgow Coma Scale (GCS) to objectively measure coma severity in all acute medical and trauma patients [1–3]. The scale measures the mental status of patients according to three categories of responsiveness: eye opening, motor response, and verbal response. Each component is assessed independently and given a score, and the sum of the scores for the three components is the GCS score. The maximum score is 15 and the minimum is 3 [2, 4]. Use of the tool became more popular in the 1980s when it was recommended by the first edition of the Advanced Trauma and Life Support for use in all trauma patients, and the World Federation of Neurosurgical Societies (WFNS) used it in its scale for grading patients with subarachnoid haemorrhage [2].

A little over four decades later, and in spite of some few criticisms such as lack of interrater reliability, the GCS continues to be the gold standard for the assessment, on-going monitoring, prognosis, and clinical judgement about consciousness in patients suffering from traumatic brain injuries and other acute neurological conditions [5, 6]. The tool is now used in over 80 countries, and elements of it have been incorporated into other assessment resources such as the early warning scoring tools, the trauma score, the revised Acute Physiology and Chronic Health Evaluation II score, and the World Federation of Neurosurgeons subarachnoid haemorrhage grading system [6, 7]. The Glasgow Coma Scale is frequently used for field triage decisions, including emergency management and the determination of a proper destination for a patient transfer. Serial Glasgow Coma Scale measurements also have value in the evaluation of the clinical course of a patient [2].

Even beyond trauma, the GCS is used routinely to monitor the clinical care of patients with virtually any condition, be it metabolic, degenerative, infective, or neoplastic, as long as it affects the nervous system directly or indirectly. It can therefore serve as a yardstick to determine whether patients are making progress in their recovery, or they are stuck at one point, or they are deteriorating [8]. In view of the broad usability of the tool across different clinical situations, it is imperative for all nurses and clinicians alike to be familiar and conversant with it, since they may need to perform a GCS on a patient at any given time [1].

Notwithstanding the simplicity and objectivity of the GCS, validity and usefulness of its scores require accurate knowledge of the tool and ability to apply it in a clinical situation. Inadequate knowledge in the application of this tool would invariably impact negatively on the care of patients with altered level of consciousness or in emergency circumstances, since deterioration in clinical status may not be readily detected until the condition becomes worse or irreversible [8].

Unfortunately, many studies that have been conducted to assess knowledge of nurses and other clinicians about the GCS have reported poor knowledge about this important tool. In a study to assess the knowledge of Nigerian physicians about the GCS, 30% of the participants did not even know the full meaning of GCS [8]. In the same country, 33% of nurses in a study to assess nurses' knowledge of the GCS had poor knowledge [9]. Similar studies conducted in Malaysia, Jordan, and Iraq also reported inadequate knowledge of the GCS among nurses [10–12]. In Vietnam, while 90% of nurses in a study were able to answer basic questions about the GCS correctly, 52.1% of the nurses answered incorrectly questions related to clinical scenarios requiring the application of the basic knowledge [13]. This means that nurses were unable to integrate their theoretical knowledge of the GCS with clinical practice. Heim, Schoettker, Gilliard, and Spahn [14] made similar findings among air-rescue physicians in Switzerland when they assessed their knowledge on the GCS and concluded that while majority of the physicians demonstrated good theoretical knowledge of the tool, many of them could not score it precisely.

This worrying trend of poor knowledge of nurses about such a life-saving and fairly simple tool in many countries calls for an assessment of the situation in Ghana, where, to the best of our knowledge, such a study has never been conducted. Our study would therefore lay the foundation for building a body of knowledge in this important area in Ghana. The study assessed nurses' knowledge about the GCS and factors associated with their knowledge.

## 2. Methods

### 2.1. Design and Setting

We conducted the study using a descriptive cross-sectional survey with a quantitative approach. In cross-sectional surveys, independent and dependent variables are measured at the same point in time [15].

The study was conducted in a large teaching hospital in the north of Ghana. It serves as a referral hospital for the three northern regions of Ghana, namely, the Northern, Upper East, and Upper West regions. It cooperates with the University for Development Studies in Northern Ghana to offer undergraduate and graduate education in medicine and allied health programs such as nursing, nutrition, medical laboratory sciences, and pharmacy [16].

As the main referral hospital in the north of Ghana, the hospital receives almost all cases of traumatic brain injuries and other complicated forms of trauma and severe medical conditions in the region, either as direct admissions or through referrals. In the year 2017, there were a total of 355 cases of traumatic brain injuries admitted to the Neurosurgical unit of the hospital [17]. These patients typically pass through the Accident and Emergency Unit of the hospital, where they are triaged and stabilized before being transferred to the Neurosurgical ward. From there, patients whose conditions remain critical are transferred to the Intensive Care Unit of the hospital for further management. Apart from these traumatic admissions, the medical wards of the hospital also admit patients with severe medical disorders with altered level of consciousness, example acute meningitis, cerebral malaria, and stroke. All these categories of patients require monitoring with the GCS. The General Surgery Department also admits and cares for surgical patients before and after surgeries. As such, the nurses in that department are also expected to monitor the level of consciousness of patients, especially during the immediate postoperative period. These characteristics make the hospital an ideal location to assess nurses' knowledge about the GCS.

### 2.2. Population and Sampling

From a population of about 160 nurses working in the five different units of the hospital where the study was conducted, a convenience sample of 115 nurses completed the survey.

The participants were chosen from three specialty wards (Accident and Emergency ward, Intensive Care Unit, and Neurosurgical ward) and two general wards (Medical ward and General surgery ward). These units were chosen because the caliber of patients in those units frequently requires monitoring with the GCS.

### 2.3. Data Collection Procedure

Following ethical approval from the Research and Ethics Committee of the Tamale Teaching Hospital, the approval letter along with consent form to introduce the study purpose and participant's rights were distributed to the Ward In-charges of all the participating wards, to inform their nurses about the study. One week after the notification, we visited the wards and administered the questionnaires to all those who had consented to participate in the study.

We then directed and supervised the participants to complete the questionnaire within 30 minutes in our presence. This was to ensure that participants did not refer to any textbooks or online resources to complete the questionnaire, which could affect the validity of the results. After a participant had finished answering, we immediately scanned the questionnaire for completeness, and then added it to other questionnaires to maintain anonymity of the participants. During data collection, we scanned each questionnaire for completeness immediately a participant finished answering, and those who did not complete any relevant portions were prompted to complete it.

### 2.4. Data Collection Instrument

The instrument for data collection was a structured questionnaire developed by the researchers. The tool comprised of three main sections designed to capture data about participants' demographic characteristics, exposure to the GCS, and knowledge about GCS.

Section “A” contained six items that asked about demographic features of participants such as age, gender, academic qualification, and current ward of practice. Section “B” had five questions about participants' exposure to the GCS like “During the course of your training in nursing, were you introduced to the GCS either in the ward or the class?” and “In your experience as a professional nurse, have you assessed and recorded the GCS of any patient?” The knowledge section of the questionnaire was section “C”, and it contained 20 multiple choice questions about the GCS derived from the literature [2]. In order to ensure validity of the tool, we pretested it on a sample of 15 nurses working in the emergency department of a large district hospital within the Tamale metropolis, and no ambiguities were found in the questions, so we maintained the questionnaire in its original form for the main study.

### 2.5. Data Analysis

The Statistical Package for Social Sciences, version 21 [18], was used to analyse data from the study. Descriptive statistics such as frequencies, percentages, and means were used to summarize the background characteristics of participants and their exposure to GCS. Knowledge of nurses on the GCS was determined from a scale of 20 multiple choice questions. Each correct answer was valued at one point, and a wrong answer attracted no point. Questions that were not answered were treated as wrong answers. A total score on knowledge was calculated for each participant from these 20 multiple choice items. Participants were then grouped into three categories based on their total score on the knowledge scale: good knowledge (80% or higher), average knowledge (60%-79%), and poor knowledge (less than 60%). We used the independent samples t-test to determine differences in knowledge between males and females, and also between those who received refresher training in GCS and those who did not. The assumption of homogeneity of variances was tested and found tenable using Levene's test* F*(113) = 1.139, p = .288. Pearson's correlations were performed to see if years of experience in the ward have an association with knowledge. The one-way ANOVA was used to determine differences in knowledge of the GCS based on the ward, academic qualification, and frequency of performing the GCS. In all cases, Levene's test for homogeneity of variances was performed and found to be untenable* F*(4, 110) = 3.784, p = .006, F(2, 112) = 1.802, p = .170, and F(3, 111) = 2.575, p = .057. Therefore, the Welch test was used to make these comparisons. An alpha value (p value) of <.05 was considered significant in all statistical tests.

### 2.6. Ethical Considerations

We obtained approval for the study from the Research and Ethics Committee of the Tamale Teaching Hospital (TTH/R&D/SR/136). Participation in the study was completely voluntary, and we made participants aware of their right to refuse to participate in the study. Those who agreed to participate signed written informed consent to confirm that they were participating voluntarily. The identity of participants remained anonymous throughout the study and we ensured confidentiality of all data.

## 3. Results

### 3.1. Background Characteristics of Participants

Many of the participants were Staff Nurses (41.7%) with more than half (55.7%) having a diploma. Most of them were in their 30s (mean age = 30.52, median 30.00, SD = 3.74) and had been working in their present wards for an average of 2.40 years. Further characteristics of the sample are presented in Table 1.

### 3.2. Exposure to the Glasgow Coma Scale

As part of identifying factors associated with participants' knowledge of the GCS, we asked them about their exposure to or previous learning experience of the GCS, and the extent to which they had performed GCS in the past. The results showed that even though 93% of the participants were taught about the GCS during the course of their training, the teaching had been very brief and superficial for 57% of the participants. Beyond their preservice training, a large majority of the participants (85.2%) had not received any refresher training on the GCS.

Further details of these findings are presented in Table 2.

### 3.3. Knowledge of the Glasgow Coma Scale

A little more than half of the participants (50.4%) in this study demonstrated poor knowledge about the GCS. Out of a possible range of scores from 0 to 20, participants had a mean score of 11.99 with a standard deviation of 3.70. Level of knowledge of participants about the GCS is presented in Table 3.

Apart from the overall knowledge of participants on the GCS as a whole, we also explored their knowledge on basic theoretical concepts about the GCS and their knowledge related to its application in clinical scenarios. For the questions on basic theoretical concepts of the GCS, majority of the participants (62.6%) demonstrated good knowledge on these items, getting 80% of the answers correct. However, when it came to questions about applying knowledge of the GCS in clinical scenarios, only 5.2% of participants were able to answer 80% of the questions correctly. Out of a possible range of scores from 0 to 10, 70.4% of the participants scored five or less, with a mean score of 4.53 and a standard deviation of 1.76. These findings are presented in Tables 4 and 5 respectively.

### 3.4. Comparison of Participants' Knowledge on GCS Based on Selected Background Characteristics

First, we tested associations between general knowledge of GCS and years of practice in the ward using Pearson's correlations, and there was no correlation between the two (p = .253). Then we examined correlation between years of experience in the ward and knowledge of the GCS in clinical scenarios, and there was no correlation between these two as well (p = .076). Next, we used the independent samples t-test to assess for differences in the level of knowledge between males and females and also between those who received refresher training on GCS and those who did not. The independent t-test showed that knowledge of females about the GCS was statistically significantly higher (12.93 ± 3.27), compared to males (11.47* ±* 3.84) (*t*(113) = 2.049, p = .043). There was no statistically significant difference in the level of knowledge between those who received refresher training on GCS and those who did not (*t*(113) = 1.222, p = .224). We also compared differences in knowledge of participants based on the ward in which they worked, their academic qualification, and frequency with which they perform the GCS. This was done using the one-way ANOVA. The ANOVA results showed that there were significant differences between groups in terms of the ward (*F*(4, 110) = 3.860, p = .001) and frequency of GCS performance (*F*(3, 111) = 4.315, p = .001). These findings are presented in Table 6.

A Games-Howell post hoc test revealed that the knowledge of nurses working in the Neurosurgical ward about the GCS was statistically significantly higher (14.20 ± 1.82) than those in the Medical ward (11.03 ± 4.71, p = .028) and General Surgery ward (10.70 ± 3.63, p = .001). Aside from this, no significant differences were observed in the level of knowledge between nurses in the other wards. Concerning the frequency of GCS performance, the Games-Howell post hoc test showed that those who perform the task weekly demonstrated statistically significantly higher levels of knowledge (15.67 ± .578) than those who perform it daily (13.34 ± 2.77, p = .005), occasionally (11.08 ± 4.10, p = .001), and almost never (11.19 ± 3.83, p = .001). Nurses who perform the GCS daily showed higher levels of knowledge than those who almost never do it (p = .017). Those who do it occasionally and those who almost never do it did not show any significant differences in their knowledge levels (p = .999). Results of the one-way ANOVA are presented in Table 6.

## 4. Discussion

This study sought to assess the knowledge of nurses about the GCS and identify factors associated with their knowledge. Consistent with previous studies, a little more than half of the participants in this study (50.4%) had poor knowledge about the GCS [10–12]. The study also revealed that while many nurses may have knowledge about the basic theoretical concepts of the GCS, they are not able to apply that basic knowledge in clinical scenarios. For instance, while 62.6% of the participants in this study demonstrated good knowledge about basic concepts of the GCS, only 5.2% of the participants had good knowledge on application of the basic knowledge in clinical situations. This finding is in tandem with the findings of Hien and Chae [13] who studied knowledge and performance of the GCS among Vietnamese nurses and reported that >90% of the participants answered correctly to questions about basic knowledge of the GCS, but 52.1% answered wrongly questions that required application of the basic knowledge in a clinical scenario. These findings suggest that nurses are not able to integrate their theoretical knowledge of the GCS with its practical application in the clinical setting. In the context of this study, the generally poor knowledge of the GCS among nurses may be due to the poor quality of teaching of the skill during preservice training, as well as lack of refresher courses on it when nurses start working. This assertion is in view of the fact that for 57.4% of the participants in this study, they were taught about the GCS during their training in a very brief and superficial manner. For another 85.2%, they had never had any refresher training on the GCS since they started working.

Concerning factors associated with nurses' knowledge of the GCS, the findings from this study confirmed earlier findings of Al-Quraan and Eid AbuRuz [10] who reported that years of practice in the ward or specialty unit were not associated with knowledge of the GCS. It however contradicts the findings of Mattar, Liaw, and Chan [19] who found that longer years of service in a neuroscience setting were associated with more knowledge about the GCS.

We however found significant association between participants' gender and their knowledge of the GCS, with female nurses demonstrating statistically significantly higher levels of knowledge than male nurses (*t*(113) = 2.049, p = .043). Similar findings were reported in an earlier study by Hien and Chae [13] where females demonstrated higher levels of knowledge than their male counterparts.

In conformity with a previous study by Ehwarieme and Anarado [9] who found higher levels of knowledge among nurses working in the Neurosurgical ward, the findings from the present study also showed that nurses in the Neurosurgical ward had the highest mean score on the knowledge scale (14.20 ± 1.82) and also had statistically significantly higher levels of knowledge than nurses in the Medical ward (11.03 ± 4.71, p = .028) and General Surgery ward (10.70 ± 3.63, p = .001). This is probably because nurses in the Neurosurgical ward frequently manage patients requiring monitoring with the GCS. One unexpected finding from this study was that nurses who performed the GCS weekly demonstrated higher levels of knowledge (15.67 ± .578) than those who performed it daily (13.34 ± 2.77, p = .005). This finding is surprising because we expected that frequent performance of the task will correlate positively with knowledge of the task, so those who perform it daily should be more knowledgeable than those who perform it weekly and so on. But this was not exactly the case in this study. It is not immediately clear what may have contributed to this finding, and further research is needed to establish the relationship between frequency of GCS performance and knowledge of the GCS.

## 5. Conclusion and Recommendations

Considering the importance of the GCS in monitoring all categories of patients with altered consciousness to detect deterioration or improvement in their condition, as established in the literature, it is quite worrying that many nurses lack adequate knowledge on the tool. It becomes more troubling when you consider the fact that this situation is not peculiar to Ghana, as studies in other countries have also reported low levels of knowledge about the GCS among nurses. There is therefore an urgent need to review how the skill is taught in nursing schools. There should be a more structured and detailed approach to teaching the skill that should go along with demonstrations.

## Figures and Tables

**Table 1:** Background characteristics of participants.

Variables	Frequency	Percentage (%) *N = 115*
*Age *		
20-24	3	2.6
25-29	43	37.4
30-34	54	47.0
35-39	12	10.4
40 and above	3	3.6

*Gender *		
Male	74	64.3
Female	41	35.7

*Position/Rank *		
Staff nurse	48	41.7
Senior staff nurse	19	16.5
Nursing officer	36	31.3
Senior nursing officer	11	9.6
Principal nursing officer	1	0.9

*Ward *		
Accident and emergency ward	22	19.1
Intensive Care Unit	14	12.2
Neurosurgical ward	20	17.4
Medical ward	26	22.6
General surgery ward	33	28.7

*Years of practice in present ward *		
1 – 3	96	83.5
4 - 6	18	15.7
7 and above	1	0.9

*Academic qualification *		
Diploma	64	55.7
First degree	44	38.3
Masters degree	7	6.1

**Table 2:** Exposure to the Glasgow Coma Scale.

Variables	Frequency	Percentage (%)
*Were you taught about the GCS during your training? *		
Yes	107	93.0
No	8	7.0
*Quality of GCS training *		
Very brief and superficial	66	57.4
Very thorough but without any demonstration	28	24.3
Very thorough with demonstrations	14	12.2
*Any refresher training on the GCS? *		
Yes	17	14.8
No	98	85.2
*Frequency of GCS performance *		
Daily	38	33
Weekly	3	2.6
Occasionally	26	22.7
Almost never	48	41.7

*Note. *Eight people (7.0%) did not answer the question on quality of GCS teaching because they had answered no in a preceding question.

**Table 3:** Knowledge levels of participants about GCS.

Knowledge level	Frequency	Percentage (%) *N = 115*
Good knowledge	21	18.3
Average knowledge	36	31.3
Poor knowledge	58	50.4

**Table 4:** Knowledge of participants on basic concepts of the GCS.

Knowledge level	Frequency	Percentage (%) *N = 115*
Good knowledge	72	62.6
Average knowledge	23	20.0
Poor knowledge	20	17.4

**Table 5:** Knowledge of participants on application of the GCS in clinical scenarios.

Knowledge level	Frequency	Percentage (%) *N = 115*
Good knowledge	6	5.2
Average knowledge	28	24.3
Poor knowledge	81	70.4

**Table 6:** Differences in GCS knowledge based on selected background characteristics.

Variables	Mean score	SD	F	p
*Ward *				
Accident and Emergency ward	12.681	3.358	3.860	.006
Intensive Care Unit	12.571	2.623		
Neurosurgical ward	14.200	1.8234		
Medical ward	11.039	4.712		
General Surgery ward	10.697	3.627		

*Academic qualification *				
Diploma	11.375	3.525	2.645	.075
First degree	12.546	3.991		
Masters degree	14.143	1.773		

*Frequency of GCS performance *				
Daily	13.342	2.773	4.315	.006
Weekly	15.667	577		
Occasionally	11.077	4.098		
Almost never	11.188	3.829		

## Data Availability

The data that was generated from this study cannot be made available to general public because of a confidentiality agreement between the researchers and participants.
